# Dynamic Crosslinking: An Efficient Approach to Fabricate Epoxy Vitrimer

**DOI:** 10.3390/ma14040919

**Published:** 2021-02-15

**Authors:** Yin Ran, Ling-Ji Zheng, Jian-Bing Zeng

**Affiliations:** Chongqing Key Laboratory of Soft-Matter Material Chemistry and Function Manufacturing, School of Chemistry and Chemical Engineering, Southwest University, Chongqing 400715, China; ranyin@email.swu.edu.cn (Y.R.); zljxndx419@emial.swu.edu.cn (L.-J.Z.)

**Keywords:** epoxy vitrimer, transesterification, dynamic crosslinking, static curing

## Abstract

Epoxy vitrimers with reprocessability, recyclability, and a self-healing performance have attracted increasingly attention, but are usually fabricated through static curing procedures with a low production efficiency. Herein, we report a new approach to fabricate an epoxy vitrimer by dynamic crosslinking in a torque rheometer, using diglycidyl ether of bisphenol A and sebacic acid as the epoxy resin and curing agent, respectively, in the presence of zinc acetylacetonate as the transesterification catalyst. The optimal condition for fabricating the epoxy vitrimer (EVD) was dynamic crosslinking at 180 °C for ~11 min. A control epoxy vitrimer (EVS) was prepared by static curing at 180 °C for ~11 min. The structure, properties, and stress relaxation of the EVD and EVS were comparatively investigated in detail. The EVS did not cure completely during static curing, as evidenced by the continuously increasing gel fraction when subjected to compression molding. The gel fraction of the EVD did not change with compression molding at the same condition. The physical, mechanical, and stress relaxation properties of the EVD prepared by dynamic crosslinking were comparable to those of the EVS fabricated by static curing, despite small differences in the specific property parameters. This study demonstrated that dynamic crosslinking provides a new technique to efficiently fabricate an epoxy vitrimer.

## 1. Introduction

Permanently crosslinked structures confer thermosets with many superior advantages, including excellent mechanical properties, chemical and thermal resistance, and dimensional stability, which makes them irreplaceable in various applications such as encapsulants [1], coatings [2], adhesives [3], electronics [4], and high performance composites [5,6,7]. However, permanently crosslinked structures also present some serious issues for thermosets [8]. On the one hand, thermosets cannot be reprocessed, reshaped, or thermally recycled once cured, leading to serious environmental concerns and resource waste at the end of their lifetime [9]. On the other hand, they cannot be fabricated or modified in regular polymer processing machines, such as an internal mixer or extruder, because they cannot melt and flow after curing [9,10,11,12,13,14]. Thus, thermosets usually possess a relatively low fabrication efficiency and poor versatility compared with thermoplastics, which can be facilely modified or produced with regular processing machines [15].

Introducing dynamic covalent bonds into crosslinked polymer networks represents a powerful approach to impart malleability and thermal processability, which are generated by network topological rearrangement as a result of an exchange reaction of dynamic covalent bonds under external stimuli, such as heat [9,16,17] and UV-light [15]. Polymer networks containing dynamic crosslinks are known as covalent adaptive networks (CANs), which are generally categorized into dissociative and associative CANs, depending on the exchange reaction mechanisms of the different dynamic covalent bonds [18]. Associative CANs, also referred to as vitrimers, have attracted increasing interest, as they retain their network structure during topological rearrangement, owning to the associative exchange reaction. The first vitrimer, based on the hydroxyl-ester exchange reaction of epoxy, was reported by Leibler and co-workers in 2011 [17]. Since then, a great deal of vitrimers have been developed based on various dynamic covalent bonds such as disulfide [19], imines [20,21,22,23], olefin metathesis [24,25,26], transamination [27,28], boronic ester [29,30,31], transesterification [11,32,33,34,35], and vinylogous urethane bonds [36]. The emergence of CANs and vitrimers makes it possible to process network polymers like linear or branched thermoplastic polymers via thermal processing techniques, which provides an efficient method for thermosets recycling [37]. Furthermore, we believe that the fabrication of crosslinked polymers could also be changed substantially by the appearance of vitrimers, because they can be post processed despite being crosslinked. For example, we reported that highly crosslinked castor oil-based poly(ester amide) networks containing disulfide bonds could be synthesized by condensation polymerization directly, and could be thermally processed into final products [38]. In contrast, castor oil-based networks without disulfide bonds could also be synthesized by the same method, but were useless because they could not be processed.

The most important and versatile thermosets are epoxy thermosets, which share ~70% of the global thermosets market and also cause a large amount of thermosetting waste [2,39,40]. As such, extensive investigations have been conducted in order to develop epoxy vitrimers through the incorporation of various dynamic covalent bonds. Most of these investigations focused on the structure design [41], malleability [42,43], and recyclability [44,45] of the vitrimers. However, the new epoxy vitrimers were still synthesized via static curing procedures similar to traditional epoxy thermosets, with a low production efficiency [15,46]. No investigations have been conducted on fabricating epoxy vitrimers through dynamic crosslinking procedures in traditional polymer processing machines, such as internal mixers or extruders. The fabrication efficiency would be improved substantially if they could be prepared this way, which is significant for the industrialization and widespread application of epoxy vitrimers.

Therefore, we evaluate the feasibility of using a dynamic crosslinking technique to synthesize epoxy vitrimer in this study. A torque rheometer (internal mixer) was used as the dynamic crosslinking equipment. An epoxy based on the hydroxyl-carboxylate exchange reaction was used as the model vitrimer, which was prepared with sebacic acid and diglycidyl ether of bisphenol A (DGEBA), with zinc acetylacetone as the exchange reaction catalyst. The structure and properties of the epoxy vitrimer prepared by dynamic curing were comparatively investigated with the counterpart synthesized by static curing in a flask.

## 2. Experimental

### 2.1. Material

DGEBA (DER 332) with an epoxy equivalent of 174 was purchased from Dow Chemical Company (Midland, TX, USA). Sebacic acid (SA; 98.5%) was obtained from Meryer Chemical Technology Co., Ltd. (Shanghai, China). Zinc acetylacetonate (Zn(acac)_2_; 99%) was bought from Micxy Chemical Co., Ltd. (Chengdu, China). All of the reagents were used directly without further purification.

### 2.2. Preparation of Epoxy Vitrimer by Dynamic Crosslinking

The dynamic crosslinking was performed in a torque rheometer (Kechuang, Shanghai, China) with a 30 mL chamber and two rollers. DGEBA (17.21 g), SA (10.00 g), and Zn(acac)_2_ (1.30 g) were added into the chamber of the torque rheometer at a predetermined temperature. The dynamic crosslinking occurred under a roller rotation rate of 50 rpm, and was monitored using the torque of the melt. The reaction was terminated after the maximum melt torque was achieved. The –COOH/epoxy equivalent ratio was 1 and Zn(acac)_2_ was 5 mol % of the carboxyl group.

### 2.3. Preparation of Epoxy Vitrimer by Static Curing

Static curing was performed in a flask with magnetic stirring (Yuhua, Zhengzhou, China) for the property comparison. DGEBA (17.21 g), SA (10.00 g), and Zn(acac)_2_ (1.30 g) were mixed by magnetic stirring at a predetermined temperature for a certain amount of time, both equal to the temperature and time used in the dynamic crosslinking.

### 2.4. Gel Fraction Measurement

The gel fraction of the sample was measured via a solvent immersing method. The product (~1 g) was immersed in chloroform for 3 days at room temperature in order to dissolve the non-crosslinked part of the sample. The insoluble part was collected by filtering and then vacuum dried at 80 °C for 24 h. The gel fraction (*G_f_*) was calculated by the following equation:

*G_f_* (%) = *W*_2_/*W*_1_ × 100% (1)

where *W*_2_ and *W*_1_ are the dry weight of insoluble part and the initial weight of the sample, respectively.

### 2.5. Fourier Transform Infrared (FT-IR) Spectroscopy

The sample film with a thickness of 0.5 mm obtained by compression molding at 180 °C for 10 min, and was used for the FT-IR measurement on a RF-5301PC spectrophotometer (Shimadzu, Kyoto, Japan). The FT-IR spectra were recorded from 4000 to 400 cm^−1^, with a resolution and scanning time of 4 cm^−1^ and 32, respectively. The FT-IR spectrum of the DGEBA (on surface of KBr sheet) was also recorded for the structure comparison.

### 2.6. Mechanical Properties

Tensile testing was performed on an E44 universal testing machine (MTS SYSTEMS (CHINA) CO., Shenzhen, China) with a crosshead speed of 10 mm/min at 40 °C). A dumbbell-shaped specimen (4 mm width and 0.5 mm thickness) was cut using a dumbbell-shaped cutter (Jingluda, Beijing, China) from the vitrimer sheet, which was prepared by compression molding at 180 °C under 20 MPa for 10 min. Five measurements were carried out for each sample and the average result was shown.

### 2.7. Stress Relaxation

Stress relaxation measurements were performed on a DHR-1 rheometer (TA Instruments, New Castle, DE, USA) using cured samples with parallel plate geometry. The diameter and thickness of the sample were 25 mm and 1 mm, respectively. An initial normal force of 5 N was used to guarantee good contact between the material and the parallel plates. After equilibration at a predetermined temperature for 5 min, a strain of 1% was applied, and the plot of storage modulus versus time was recorded for the stress relaxation analysis.

### 2.8. Dynamic Mechanical Analysis (DMA)

Dynamic mechanical properties were investigated using a instrument DMA1 (Mettler Toledo, Zurich, Switzerland) in a tensile mode from −40 to 120 °C, at a heating rate of 3 °C/min and an oscillation frequency of 1 Hz.

## 3. Results and Discussion

### 3.1. Epoxy Vitrimer Fabrication

**Scheme 1:**
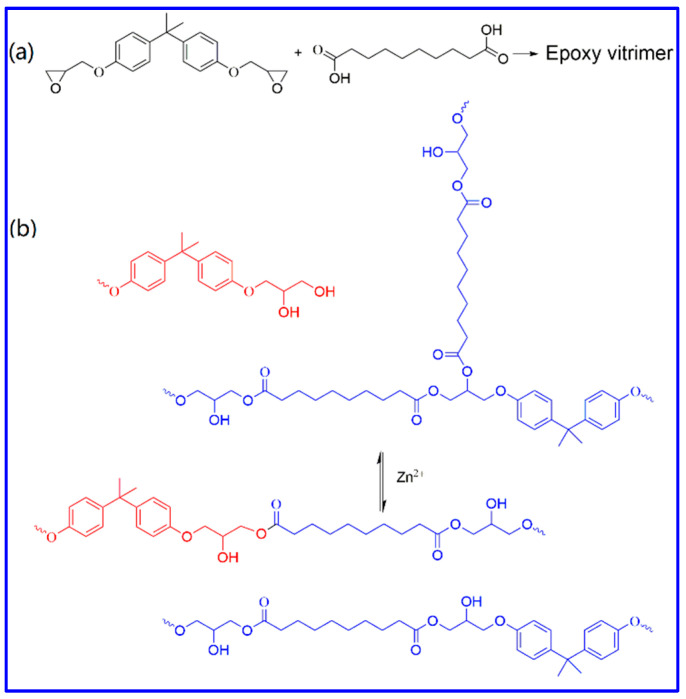
Preparation of an epoxy vitrimer from diglycidyl ether of bisphenol A (DGEBA), sebacic acid (SA), and Zn(acac)_2_ (**a**); Zn^2+^ catalyzed transesterification induced the network topological rearrangement of the epoxy vitrimer (**b**).

The epoxy vitrimer, based on transesterification, was prepared from DEGBA with SA as a curing agent and Zn(acac)_2_ as a transesterification catalyst, as shown in Scheme 1. The network topological rearrangement took place via a Zn^2+^ accelerated exchange reaction between the hydroxyl and ester group at elevated temperatures (Scheme 1b). As motioned in the Introduction, such an epoxy vitrimer is traditional fabricated through static curing in glass reactors. Herein, we evaluated the possibility of using dynamic crosslinking to fabricate the epoxy vitrimer in a torque rheometer.

**Figure 1:**
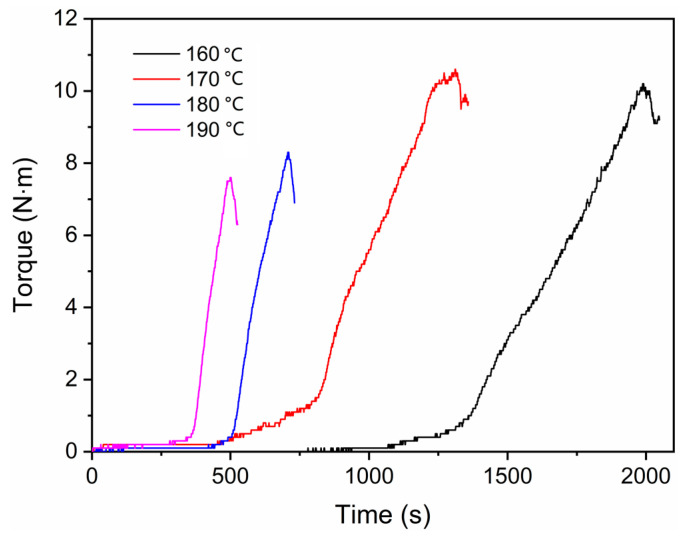
Development of torque over time for the dynamic crosslinking of diglycidyl ether of bisphenol A (DGEBA) with sebacic acid (SA) in the presence of Zn(acac)_2_, carried out at different temperatures.

Figure 1 shows the development of the torque with time for the dynamic crosslinking of DGEBA with SA in the presence of Zn(acac)_2_, carried out at different temperatures. The torque maintained a low value and almost held steady in the early stage, corresponding to the formation of low molecular weight oligomers. As the reaction proceeded, the torque began to increase sharply with the increase in time, because of the formation of crosslinked epoxy vitrimers. The torque reached the maximum value quickly and then decreased with the further increase in time. We found that the homogeneous melts changed to particle-like substances at this stage, attributed to the shearing of the rollers on the crosslinked epoxy vitrimers. We terminated the dynamic crosslinking once the torque began to decrease.

The reaction temperature mainly affected the dynamic crosslinking process. The time required for the sample to reach maximum torque decreased with the increase in temperature, because of the activated curing rate of the SA-cured DGEBA system at elevated temperatures. It took 33.2 min for the sample to reach maximum torque at 160 °C. The time reduced to 21.6, 11.1, and 8.4 min as the temperature increased to 170, 180, and 190 °C, respectively. It was found that the maximum torque decreased gradually with the increasing temperature, which is reasonable, because polymers possess a higher segment mobility, and thus a lower stiffness and lower melt torque at a higher temperature. The gel fractions were measured to be 79.3%, 81.5%, 85.9%, and 79.4% for the four samples prepared at 160, 170, 180, and 190 °C, respectively, indicating that the network structures were obtained by dynamic crosslinking, regardless of the reaction temperature considered herein. It is noted that the gel fraction increased first and then decreased with the increasing temperature. The maximum gel fraction occurred at 180 °C, possibly indicating that 180 °C is the optimal temperature for preparation of an epoxy vitrimer using DGEBA and SA through dynamic crosslinking. When the temperature increased to 190 °C, although the time required to complete the curing decreased, the gel fraction of the vitrimer decreased compared with the sample prepared at 180 °C, which is possibly because the thermal degradation occurred because of the presence of an ester bond formed by curing DGEBA with sebacic acid.

**Figure 2:**
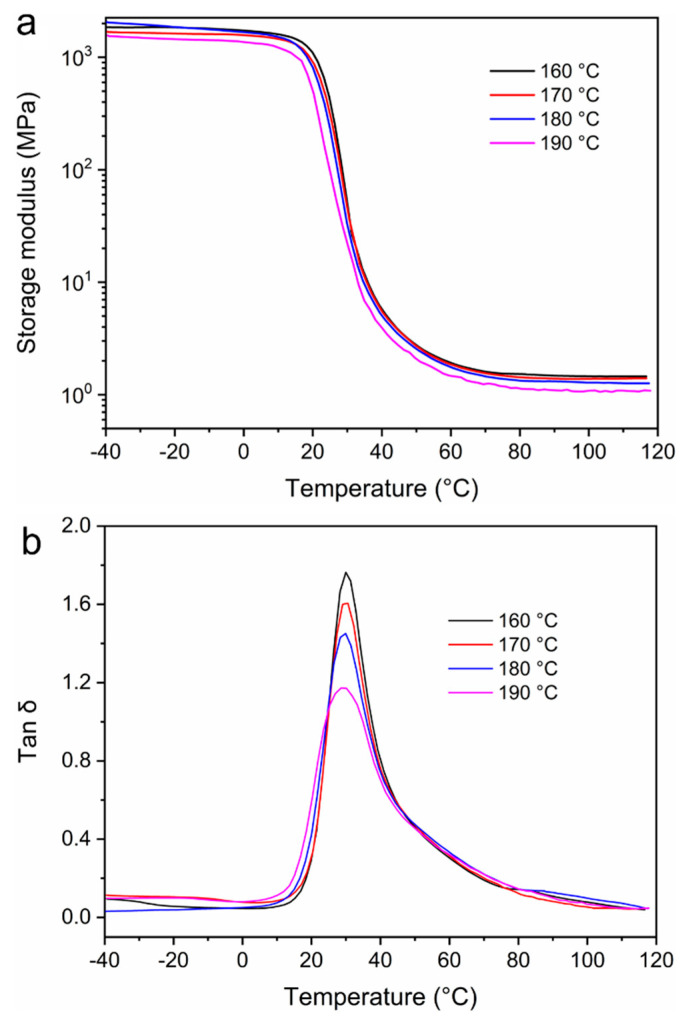
(**a**) Storage modulus plots and (**b**) tan δ plots of epoxy vitrimers prepared by dynamic crosslinking at different temperatures.

The thermal mechanical properties of the samples prepared at different temperatures were studied by DMA, as shown in Figure 2. The storage moduli of the samples prepared at 160–180 °C were close to each other and were higher than that of the sample prepared at 190 °C in the full temperature range. In addition, the glass transition temperature (*T*_g_) of the vitrimer prepared at 190 °C (28.6 °C) obtained from the tan δ plot was also slightly lower than those of the samples prepared at 160–180 °C (~29.9 °C). These phenomena should correspond to the possible thermal degradation of the sample prepared by dynamic crosslinking at a temperature greater than 180 °C, such as 190 °C. Therefore, 180 °C is considered the optimal temperature for the dynamic crosslinking of DGEBA with SA, in the presence of Zn(acac)_2_ as the transesterification catalyst.

**Figure 3:**
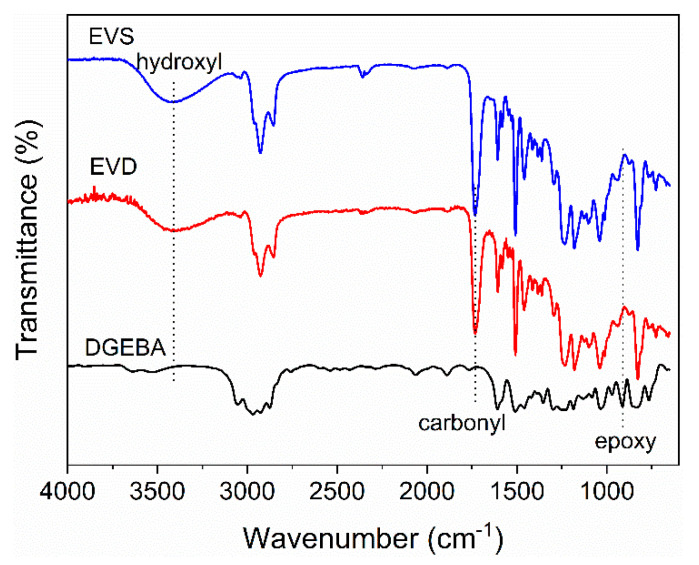
FT-IR spectra of DGEBA, epoxy vitrimers prepared by dynamic crosslinking (EVD), and epoxy vitrimers prepared by static curing (EVS).

For the property comparison, a control epoxy vitrimer was also prepared by static curing of DGEBA and SA with Zn(acac)_2_ in a flask at 180 °C for 11.1 min. For brevity, the epoxy vitrimers prepared by static curing and dynamic crosslinking at 180 °C were designated as EVS and EVD, respectively. The gel fraction of EVS was 84.4%, which was slightly lower than that of EVD, with a gel fraction of 85.9%. The chemical structures of EVD and EVS were characterized by FT-IR spectra and were compared with that of DGEBA, as shown in Figure 3. The characteristic absorption belonging to the epoxy group of DGEBA at 915 cm^−1^ disappeared completely in the spectra of EVD and EVS, indicating that the epoxy group was consumed completely by either dynamic crosslinking or static curing at 180 °C for 11.1 min. Furthermore, two new absorption bands appeared at 3422 and 1733 cm^−1^ in the spectra of both EVD and EVS, attributed to the formation of hydroxyl and ester carbonyl from the ring opening reaction between DGEBA and SA.

### 3.2. Mechanical Properties

**Figure 4:**
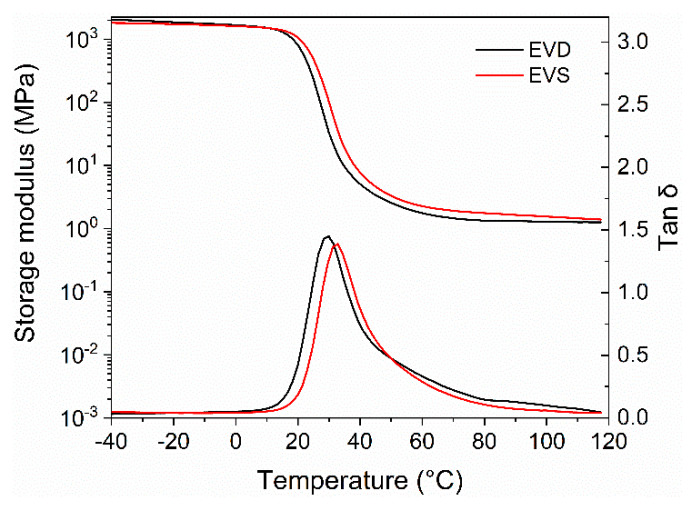
Storage modulus plots and tan δ plots of EVD and EVS.

The dynamic mechanical properties of the samples were investigated using DMA. Figure 4 shows the storage modulus plots and tan δ plots of EVD and EVS. It can be observed that EVS showed a slightly higher storage modulus than EVD at a temperature higher than the α transition, and a relatively lower peak height during the α transition in the tan δ plots. The glass transition temperature (*T*_g_; peak temperature of the tan δ plots) of EVD (29.9 °C) was slightly lower than that of EVS (32.8 °C). To gain insight into the differences in the dynamic mechanical properties of the two samples, we measured the gel fraction of the samples after compression molding at 180 °C under 20 MPa for 10 min, and found that the gel fraction of EVS increased from 84.4% to 90.6%, while that of EVD remained almost unchanged with a value of 85.9% before and after compression molding. These phenomena indicated that the curing reaction did not finish completely after static curing at 180 °C for 11.1 min, and thus the curing proceeded during a compression molding process, leading to a higher gel fraction. In contrast, the curing reaction took place almost entirely by dynamic crosslinking at 180 °C for 11.1 min, as evidenced by the remaining gel fraction after compression molding. With a relatively higher gel fraction, the EVS showed a relatively lower segment mobility, and thus a lower damping peak height during the transition zone, as well as a higher storage modulus at a temperature higher than *T*_g_.

**Figure 5:**
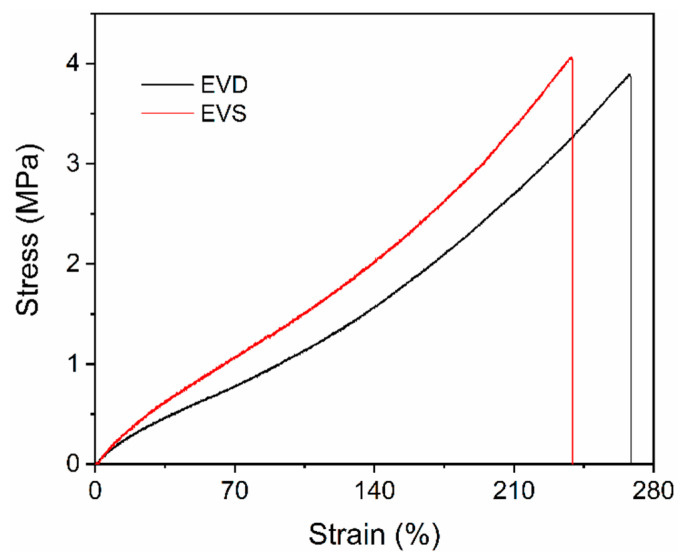
Stress-strain curves of EVD and EVS recorded at 40 °C.

The static mechanical properties of the EVD and EVS were comparatively investigated through tensile testing, as shown in Figure 5. Sample sheets with a thickness of 0.5 mm were prepared by compression molding at 180 °C under 20 MPa for 10 min. Dumbbell-shaped specimens with a width of 4 mm were cut from the sample sheets for the tensile testing. As the glass transition temperatures of the sample were close to room temperature. We conducted the tensile testing at the rubbery state (40 °C) of the samples so as to exclude the influence of the different *T*_g_s of the samples. Figure 5 shows the stress–strain curves of EVD and EVS recorded at 40 °C. Both EVD and EVS showed the typical tensile behavior of elastomers, without obvious yielding. The tensile strength, Young’s modulus, and elongation at the break of EVD were 3.56 ± 0.31 MPa, 1.26 ± 0.11 MPa, and 270 ± 1%, respectively. In contrast, the tensile strength, Young’s modulus, and elongation at the break of EVS were 4.22 ± 0.51 MPa, 1.78 ± 0.19 MPa, and 223 ± 20%, respectively. The small differences in the mechanical property parameters were attributed to the different degrees of crosslinking of the samples prepared after compression molding. The EVS with a higher gel fraction exhibited a higher tensile strength and Young’s modulus, but lower elongation at break in comparison with EVD.

### 3.3. Stress Relaxation

**Figure 6:**
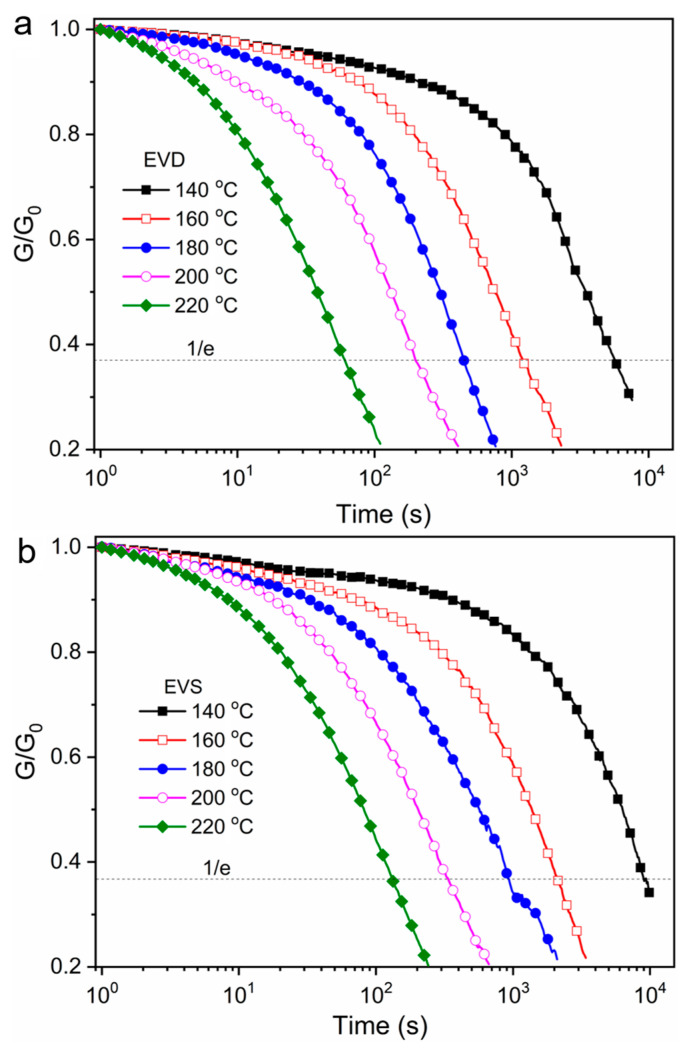
Stress-relaxation curves of (**a**) EVD and (**b**) EVS at different relaxation temperatures.

The disk-like specimens obtained by compression molding of the as-prepared epoxy vitrimer samples at 180 °C under 20 MPa for 10 min were used for the stress-relaxation measurements. Figure 6 shows the stress-relaxation curves of EVS and EVD at temperatures ranging 140–220 °C. Obvious relaxation occurred for both samples, because of the Zn^2+^ catalyzed transesterification induced topological rearrangement of the epoxy network. The relaxation was accelerated with the increasing temperature, which was attributed to the activated transesterification at elevated temperatures. The relaxation time (τ), referring to the time needed to relax the initial stress to 1/e, was used for the quantitative analysis. The τ values for the relaxation of EVS at 140, 160, 180, 200, and 220 °C were 155.5, 35.0, 15.5, 5.6, and 2.2 min, respectively. The corresponding τ values for the EVD counterparts were 95.1, 20.1, 7.5, 3.3, and 1.0 min, respectively. The results indicate that EVD possessed a faster relaxation rate. The higher gel fraction (i.e., degree of crosslinking) of EVS after compression molding, as discussed above, should account for the slower relaxation rate in comparison with EVD.

The relaxation time τ at different temperatures (*T*) can be analyzed with Arrhenius equation, as follows:

τ(*T*) = τ_0_ exp(*E_a_*/R*T*) (2)

**Figure 7:**
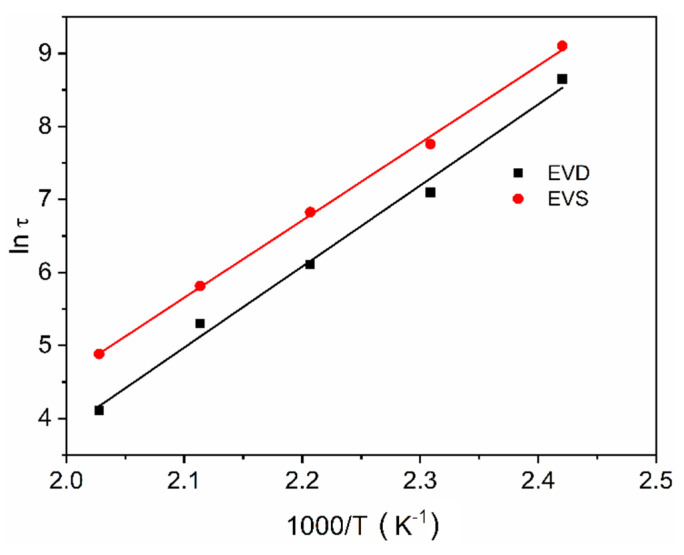
Linear fitting of the relaxation time versus temperature according to the Arrhenius equation for relaxation activation energy determination.

As Figure 7 shows, two straight lines were obtained through the linear fitting of the relaxation time versus temperature, indicating that the Arrhenius equation could indeed describe the relaxation of the epoxy vitrimers based on Zn^2+^ catalyzed transesterification. The relaxation activation energy *E_a_* of EVD was calculated to be 88.07 kJ/mol, which is slightly lower than that of EVS, with the value of 92.29 kJ/mol. The relatively higher *E_a_* for EVS was also attributed to its relatively higher degree of crosslinking after compression molding, which led to decreased relaxation.

## 4. Conclusions

We demonstrated a new approach for fabricating an epoxy vitrimer based on Zn^2+^ catalyzed transesterification by dynamic crosslinking in a torque rheometer. Because of the capability of the topological rearrangement of the epoxy vitrimer network, the as-prepared epoxy vitrimer could be thermally processed with traditional processing techniques such as compression molding. The physical, mechanical, and stress relaxation properties of the epoxy vitrimer prepared by dynamic crosslinking were comparable to the counterpart prepared by static curing in a flask. We believe that this technique can be extended to the fabrication of other vitrimers, and should also be suitable for the preparation of vitrimers containing polymer blends and composites, as the high stress of the processing machine can facilitate the dispersion of the components. Furthermore, this technique is meaningful for the industrialization of epoxy vitrimers, because it can easily be scaled up.

## Data Availability

Data sharing not applicable.
